# Inhibition of α_1_-Adrenergic, Non-Adrenergic and Neurogenic Human Prostate Smooth Muscle Contraction and of Stromal Cell Growth by the Isoflavones Genistein and Daidzein

**DOI:** 10.3390/nu14234943

**Published:** 2022-11-22

**Authors:** Ru Huang, Yuhan Liu, Sheng Hu, Alexander Tamalunas, Raphaela Waidelich, Frank Strittmatter, Christian G. Stief, Martin Hennenberg

**Affiliations:** Department of Urology, University Hospital, LMU Munich, 81377 Munich, Germany

**Keywords:** benign prostatic hyperplasia (BPH), lower urinary tract symptoms (LUTSs), voiding symptoms, soy, soy food, isoflavones, prostate smooth muscle contraction

## Abstract

Isoflavone-rich legumes, including soy, are used for food production, as dietary supplements and in traditional medicine. Soy consumption correlates negatively with benign prostatic hyperplasia (BPH) and voiding symptoms. However, isoflavone effects on the prostate are hardly known. Here, we examined the effects on human prostate smooth muscle contractions and stromal cell growth, which are driving factors of voiding symptoms in BPH. Smooth muscle contractions were induced in prostate tissues from radical prostatectomy. Growth-related functions were studied in cultured stromal cells (WPMY-1). Neurogenic, α_1_-adrenergic and non-adrenergic contractions were strongly inhibited with 50 µM and by around 50% with 10 µM genistein. Daidzein inhibited neurogenic contractions using 10 and 100 µM. Agonist-induced contractions were inhibited by 100 µM but not 10 µM daidzein. A combination of 6 µM genistein with 5 µM daidzein still inhibited neurogenic and agonist-induced contractions. Proliferation of WPMY-1 cells was inhibited by genistein (>50%) and daidzein (<50%). Genistein induced apoptosis and cell death (by seven-fold relative to controls), while daidzein induced cell death (6.4-fold) without apoptosis. Viability was reduced by genistein (maximum: 87%) and daidzein (62%). In conclusion, soy isoflavones exert sustained effects on prostate smooth muscle contractions and stromal cell growth, which may explain the inverse relationships between soy-rich nutrition, BPH and voiding symptoms.

## 1. Introduction

Genistein, daidzein and puerarin are isoflavones naturally occurring in plants of the *Fabaceae* family, including soybeans (*Glycine max*), red clover (*Trifolium pratense*) and different *Pueraria* species, the latter known as Kudzu plant in Western nations and as Gegen in China [1,2]. Based on observations from traditional medicine and of soy consumption for nutrition, as well as epidemiological, experimental and clinical studies, a plethora of biological effects have been attributed to isoflavones, including antitumorigenic, antidiabetic, neuroprotective, cardioprotective, estrogenic and hormone-disrupting effects, in addition to vasorelaxation and inhibition of smooth muscle contractions [2,3]. In soy and red clover, genistein and daidzein are predominant isoflavones and constitute major parts of the total isoflavone pool [1,4]. In *Pueraria* species, the predominant isoflavone is puerarin [1,4]. Isoflavone-rich preparations from soy and red clover and from genistein itself are available as dietary supplements and used against menopause symptoms or to reduce cholesterol levels [5,6,7]. Preparations from different *Pueraria* species are used in Western countries (“Kudzu”) and in traditional medicines in China (“Gegen”) and other Eastern Asian regions for the treatment of cardiovascular diseases, diabetes, liver injury and symptoms of diarrhea and fever, as well as to improve circulation and blood flow [8,9,10,11].

Soy-rich nutrition has been repeatedly associated with different health-promoting effects, including improvement of benign prostatic hyperplasia (BPH) and lower urinary tract symptoms (LUTSs). In epidemiological studies, consumption of soy food correlated negatively with the prevalence of BPH and LUTSs, which was supposed to account for the low prevalences of BPH in Eastern Asian regions with traditionally high soy consumption rates [12,13,14]. Increasing isoflavone exposure, resulting from the increased popularity and consumption of soy food that has been noted in Western countries, has unknown risks and benefits, raising the need for improved understanding of isoflavone effects [15].

Voiding symptoms in BPH are characterized by impaired urinary flow and bladder emptying due to urethral obstruction driven by increased prostate smooth muscle tone, enlargement of the prostate or both [16,17]. Accordingly, contraction and growth in the prostate are targets for medical treatment in BPH. Available drugs include α_1_-adrenoceptor antagonists (α_1_-blockers) and the phosphodiesterase 5 inhibitor tadalafil for rapid symptom improvements by smooth muscle relaxation and 5α-reductase inhibitors to reduce prostate growth, progression and the risk of complications and surgery [18]. However, the average improvements by available medications do not exceed 50%, which level is not far from that of placebos and contributes to dissatisfaction, discontinuation and finally hospitalization and surgery [18,19]. Combination therapies are required for simultaneous symptom improvements and prevention of progression but are beset by discontinuation rates of up to 90% [20]. The limitations of α_1_-blockers, which are the first-line option for medical treatment of male LUTSs, have been explained by non-adrenergic contractions, which may raise maximum smooth muscle tone in the prostate and could maintain urethral obstruction in medication-refractory LUTSs [21,22].

While relationships between isoflavone-rich nutrition and BPH/LUTSs have been widely assumed and anticontractile effects have been repeatedly reported in different smooth-muscle-rich organs, isoflavone effects in the prostate are poorly known. Previous studies addressing their effects on prostate smooth muscle contraction have not included human tissues, being limited to non-human prostates. In parallel, attempts to address isoflavone effects on the growth of prostate cells have largely focused on glandular cells. Here, we speculated that leguminous isoflavones may inhibit agonist-induced and neurogenic contractions of human prostate smooth muscle and affect the growth-related functions of stromal cells. Thus, the aim of this study was to examine the effects of genistein, daidzein and puerarin on α_1_-adrenergic, non-adrenergic and neurogenic contractions of human prostate tissues and on growth-related functions of cultured stromal cells.

## 2. Materials and Methods

### 2.1. Human Prostate Tissues

Prostate tissues were obtained from patients undergoing radical prostatectomy for prostate cancer. This study was performed in accordance with the Declaration of Helsinki of the World Medical Association and was approved by the Ethics Committee of the Ludwig-Maximilians University, Munich, Germany. Informed consent was obtained from all patients. All samples and data were collected and analyzed anonymously. Accordingly, no patient data were collected, stored or analyzed in the context of this study, and samples were not grouped for pathological backgrounds or any other condition. Macroscopic inspection of prostates for tumor burden and subsequent sampling were performed by pathologists, approximately within 30 min following the final resection of prostates from patients. Organ bath experiments were started within 60 min following sampling. For transport and storage, organs and tissues were stored in Custodiol^®^ solution (Köhler, Bensheim, Germany). For macroscopic examination and sampling, the prostate was opened by a single longitudinal cut from the capsule to the urethra. Subsequently, both intersections were checked macroscopically for any obvious tumor infiltration. Tissues were taken solely from the transitional, periurethral zone, while most prostate cancers arise in the peripheral zone [23,24]. In fact, tumor infiltration in the periurethral zone (where sampling was performed) was found in less than 1% of prostates. Prostates showing tumors in the periurethral zone upon macroscopic inspection were not subjected to sampling. BPH is present in ca. 80% of patients with prostate cancer [25,26]. The average age of patients undergoing radical prostatectomy in our department is 66 ± 7 years [27], where the prevalence of histological BPH may range between 60 and 70% [16]. Typically, tissues sampled under the same conditions in our previous studies showed prostate-characteristic architectures with compositions of glands and smooth-muscle-rich stroma [28], while tissues containing only stroma without glands were usually limited to anterior parts of the human prostate [29].

### 2.2. Organ Bath

Strips of prostate tissues (6 × 3 × 3 mm) were mounted in chambers of an organ bath system (myograph 720 M, Danish Myotechnology, Aarhus, Denmark). The device includes four chambers, each filled with 10 mL aerated (95% O_2_ and 5% CO_2_) Krebs–Henseleit solution (37 °C, pH 7.4) with the following composition: 118 mM NaCl, 4.7 mM KCl, 2.55 mM CaCl_2_, 1.2 mM KH_2_PO_4_, 1.2 mM MgSO_4_, 25 mM NaHCO_3_, 7.5 mM glucose. After mounting, tissues were stretched to a tension of 4.9 mN and equilibrated for 45 min. Typically, pretensions decrease spontaneously at the beginning of the equilibration period. Therefore, tensions were adjusted three times during the equilibration period, until a stable resting tone of 4.9 mN was attained within 45 min. Subsequently, contractions induced by 80 mM KCl were assessed by the addition of a 2 M stock solution to assess highmolar KCl-induced contractions as a measure of smooth muscle content and condition and as a later reference for agonist-induced and neurogenic contractions. Highmolar KCl results in biphasic responses, including a phasic contraction reaching a peak within a few minutes after the addition of KCl, which was used for normalization, as described below, followed by a decline to a tonic, stable contraction [30]. After the peak of the phasic contraction was obtained and the tension started to decline to the tonic phase, the chambers were washed by replacing the Krebs–Henseleit solution three times, resulting in a stable resting tone close to the first baseline before KCl. Thereafter, genistein, daidzein and puerarin, or equivalent volumes of dimethylsulfoxide (DMSO, solvent for genistein and daidzein) or methanol (solvent for puerarin) as controls were added. Cumulative concentration–response curves for agonists or frequency response curves by electric field stimulation (EFS) were created 30 min after the addition of isoflavones or solvent.

EFS simulates action potentials, resulting in smooth muscle contractions of prostate tissues by release of endogenous neurotransmitters, including noradrenaline. Using tetrodotoxin, it has been demonstrated that neurotransmission accounts for two-thirds or more of EFS-induced contractions in the human prostate [31,32]. For EFS, tissue strips were placed between two parallel platinum electrodes connected to a CS4 stimulator (Danish Myotechnology, Denmark). Square pulses with durations of 1 ms were applied with a voltage of 20 V for a train duration of 10 s. EFS-induced contractile responses were studied at frequencies of 2, 4, 8, 16 and 32 Hz, with train intervals of 30 s between stimulations.

For a single independent experiment, all four chambers of one organ bath device were filled with tissues from the same prostate. Two of them were examined with isoflavone and the two others with solvent for the controls. Only one frequency response or concentration–response curve was recorded with each mounted tissue. Allocation of isoflavones and solvent to the different chambers was randomly changed between experiments. Independent experiments were repeated in indicated numbers (n), using tissues from n different patients, resulting in numbers of independent experiments as indicated for each series. Single experiments were based on double determinations wherever this was possible. From a total of 200 organ bath experiments, double determinations for both groups (i.e., control and drug groups, each with two tissues from the same prostate) were possible in 164 experiments. In the remaining experiments, the number of sampled tissues did not allow the filling of two channels for both groups. Thus, in 22 experiments, double determinations were possible in only one of the two groups, while 14 experiments were performed with only one tissue for both groups. However, each experiment contained at least one sample for both groups, resulting in paired samples.

For the calculation of agonist- or EFS-induced contractions, tensions (peak heights in EFS-induced contractions and maximum contractions following agonist exposure) were expressed as % of 80 mM KCl-induced contractions (maximum of phasic contraction). Normalization to KCl may correct variations in smooth muscle content, tissue composition and stromal/epithelial ratios, resulting from varying phenotypes and degrees of BPH, or from other individual heterogeneities between samples and patients. For calculation, the maximum contraction level at a given agonist concentration or given frequency was assessed, regardless of the pattern of contraction. In fact, contraction patterns in our samples differed between α_1_-adrenergic and non-adrenergic agonists and between tissues and patients [30]. In either case, however, the next higher concentration in the concentration–response curves was applied as soon as the plateau contraction was attained and no further substantial increase was expected. Contractions in concentration and frequency response curves describe the maximum contraction levels at each concentration and frequency in control and drug groups.

E_max_ values, EC_50_ values for contractile agonists and frequencies (f) inducing 50% of the maximum EFS-induced contraction (Ef_50_) were calculated for each single experiment by curve fitting using GraphPad Prism 6 (Statcon, Witzenhausen, Germany) and analyzed as described below. As presentation of single values in scatter plots was intended, curve fitting was performed separately for each single experiment, resulting in separate values for each independent experiment. Curves were fitted without predefined constraints for bottom, top or EC_50_ values, by ordinary fit, without weighting and without choosing automatic outlier elimination by non-linear regression. The resulting values were checked for plausibility, and settings were adapted as follows if error messages occurred, as recommended in the “GraphPad Curve Fitting Guide” (GraphPad Software Inc., San Diego, CA, USA). Thus, high concentrations were excluded in two experiments with endothelin-1 because the results were marked as “ambiguous”, as full contractions were obtained already at low concentrations in one experiment or the curve could not be converged due to downhill parts at higher concentrations. 

### 2.3. Cell Culture

WPMY-1 cells are an SV40 large-T antigen-immortalized cell line, obtained from the stroma of a human prostate without prostate cancer [33]. According to the typical composition of the prostate stroma, where smooth muscle cells are the predominant cell type, WPMY-1 cells show characteristics of myofibroblasts and prostate smooth muscle cells, including expression of vimentin, α-smooth muscle actin, calponin and α_1A_-adrenoceptors, but lacking expression of cytokeratins and tyrosine hydroxylase [28,33,34]. WPMY-1 cells were purchased from the American Type Culture Collection (ATCC, Manassas, VA, USA) and cultured in RPMI 1640 (Gibco, Carlsbad, CA, USA) supplemented with 10% fetal calf serum (FCS) and 1% penicillin/streptomycin at 37 °C with 5% CO_2_. Before the addition of isoflavones, DMSO (solvent for genistein and daidzein) or methanol (solvent for puerarin) to the cells, the medium was changed to an FCS-free medium.

### 2.4. Proliferation Assay

The proliferation rate of cells was assessed using the 5-ethynyl-2′-deoxyuridine (EdU)-based EdU-Click 555 proliferation assay kit (Baseclick, Tutzing, Germany), according to the manufacturer’s instructions. In this assay, incorporation of EdU into the DNA of proliferating cells is assessed by detection with fluorescing 5-carboxytetramethylrhodamine (5-TAMRA). Thirty thousand cells were placed in each well of a 16-well chambered coverslip (Thermo Scientific, Waltham, MA, USA) and cultured for 24 h. Subsequently, the medium was replaced by 10 mM EdU solution in FCS-free smooth muscle cell medium containing isoflavones or solvent, and, 24 h later, the cells were fixed with ROTI^®^ Histofix 4% solution (Roth, Karlsruhe, Germany). Counterstaining of all nuclei was performed with DAPI. Finally, analysis was performed by fluorescence microscopy (excitation: 546 nm; emission: 479 nm) using a laser scanning microscope (Leica SP2, Wetzlar, Germany). Stainings were quantified using “ImageJ” (National Institutes of Health, Bethesda, AR, USA).

### 2.5. Cell Apoptosis and Cell Death Analysis

A flow-cytometry-based annexin V allophycocyanin (APC) and 7-aminoactinomycin D (7-AAD) apoptosis detection kit (BD Biosciences, Franklin Lakes, NJ, USA) was used to detect apoptotic (annexin V-positive, 7-AAD-negative) and dead (annexin V-positive, 7-AAD-positive) cells. Cell death in annexin V-positive/7-AAD-positive cells may result from apoptosis or necrosis, which cannot be distinguished by this assay. Cells were grown in 6-well plates, and isoflavones or solvent were added 24 h before flow cytometry. The remaining medium of each well was collected and centrifuged to collect the cell debris. Subsequently, the cells were washed with PBS and resuspended (together with the corresponding debris collected) in annexin V binding buffer (BD Biosciences), followed by the addition of 5 μL APC annexin V and 5 μL 7-AAD reagent to every 10^5^ cells from each sample. After incubation in the dark for 15 min at room temperature, 400 μL binding buffer was added to each sample before analysis by flow cytometry.

### 2.6. Viability Assay

Viability was assessed using the Cell Counting Kit-8 (CCK-8) (Sigma-Aldrich, Munich, Germany). Cells were seeded in 96-well plates (5000 cells/well) and cultured for 24 h. Subsequently, isoflavones or solvent in required amounts were added, and cells were cultured for further 24, 48 or 72 h until assessment. Finally, 10 μL of [2-(2-methoxy-4-nitrophenyl)-3-(4-nitrophenyl)-5-(2,4-disulfophenyl)-2H-tetrazolium monosodium salt (WST-8) from the kit were added, and absorbance in each well was measured at 450 nm after incubation for 2 h at 37 °C.

### 2.7. Drugs and Nomenclature

Genistein (5,7-dihydroxy-3-(4-hydroxyphenyl)-4H-1-benzopyran-4-one), daidzein (7-hydroxy-3-(4-hydroxyphenyl)-4H-1-benzopyran-4-one) and puerarin (8-(β-D-glucopyranosyl-7-hydroxy-3-(4-hydroxyphenyl)-4H-1-benzopyran-4-on) are isoflavones naturally occurring in soybeans (*Glycine max*), red clover (*Trifolium pratense*) and Kudzu (*Pueraria* spp.). Synthetic genistein (G6649), synthetic daidzein (D7802) and puerarin (P5555, lot no. MKCG6104) were purchased from Sigma-Aldrich (Munich, Germany). Stock solutions (10 mM) of genistein and daidzein were prepared with DMSO and with puerarin using methanol (as recommended by the providers), and all were stored at −20 °C until use.

Phenylephrine ((R)-3-[-1-hydroxy-2-(methylamino)ethyl]phenol) and methoxamine (α-(1-aminoethyl)-2,5-dimethoxybenzyl alcohol) are α_1_-selective adrenoceptor agonists. Aqueous stock solutions (10 mM) of noradrenaline, phenylephrine and methoxamine were freshly prepared before each experiment. Aqueous stock solutions of endothelin-1 (0.4 mM) were stored at −20 °C as small aliquots, so that repeated freezing and thawing cycles were avoided. U46619 ((Z)-7-[(1S,4R,5R,6S)-5-[(E,3S)-3-hydroxyoct-1-enyl]-3-oxabicyclo [2.2.1]heptan-6-yl]hept-5-enoic acid) is an agonist of the thromboxane A_2_ receptor and was dissolved in ethanol. Stock solutions (10 mM) were stored at −80 °C until use. U46619 and endothelin-1 were obtained from Enzo Life Sciences (Lörrach, Freiburg im Breisgau, Germany). Noradrenaline, phenylephrine and methoxamine were obtained from Sigma-Aldrich (Munich, Germany).

### 2.8. Statistical Analyses

Data in the frequency and concentration–response curves are means ± standard deviations (SDs). E_max_, Ef_50_ and EC_50_ values are presented as single values (i.e., means from double determinations, as described above) from all independent experiments in scatter plots. Effect sizes become obvious from concentration–response and frequency response curves and scatter plots. Changes in EC_50_ values are additionally reported as mean differences (MDs) with 95% confidence intervals (Cis) in the text if the effects were obvious. Changes in E_max_ values observed in contraction experiments and cell culture experiments are reported as percentage decreases or x-fold increases (means with 95% CIs) in the text as well and were calculated by referring isoflavone samples to the corresponding controls (set to 100% or to one) in each single experiment. Calculation of MDs with 95% CIs was performed using GraphPad Prism 6. Derived from our experimental design, the control and drug groups were paired in each series. Comparison of whole frequency/concentration–response curves, i.e., of both groups within a series, was performed by two-way analysis of variance (ANOVA). Post hoc analyses for comparisons at single-agonist concentrations or frequencies were not performed, as this is discouraged in the “GraphPad Statistics Guide” (GraphPad Software Inc., San Diego, CA, USA). E_max_, Ef_50_ and EC_50_ values were compared by paired Student’s *t*-tests. All statistical tests were performed using GraphPad Prism 6. *p*-values < 0.05 were considered significant.

The present study and analyses have an exploratory character, as important features of hypothesis-testing studies are lacking [35]. Owing to the exploratory study design, the *p*-values reported here need to be considered as descriptive; they are not hypothesis-testing [35]. In order to use *p*-values sparingly [35], no *p*-values are reported in the text and *p*-values of 0.05 or higher are not indicated.

The minimum number of independent experiments in each series was pre-planned as *n* = 5, as statistical analyses with group sizes <5 have been discouraged [36]. Data were analyzed after five experiments in a series were completed, with the exception of the flow cytometry experiments, where six experiments were performed for each series. Originally, we intended to decide whether a series was to be continued or not following this interim analysis. This procedure is possible, as our study was explorative and not designed to test a pre-specified statistical null hypothesis [35], and flexible group sizes have been recommended if data are characterized by large variations, which applies here [36]. However, all series in this study revealed conclusive findings after five independent experiments or six initial independent experiments in the case of flow cytometry. For technical reasons, six instead of five experiments were performed for flow cytometry. In all series, no experiments were excluded from the analyses, and no data were excluded, with the required exceptions for curve fitting in two experiments, as described above.

## 3. Results

### 3.1. Effects of Genistein on α_1_-Adrenergic Contractions

**Figure 1:**
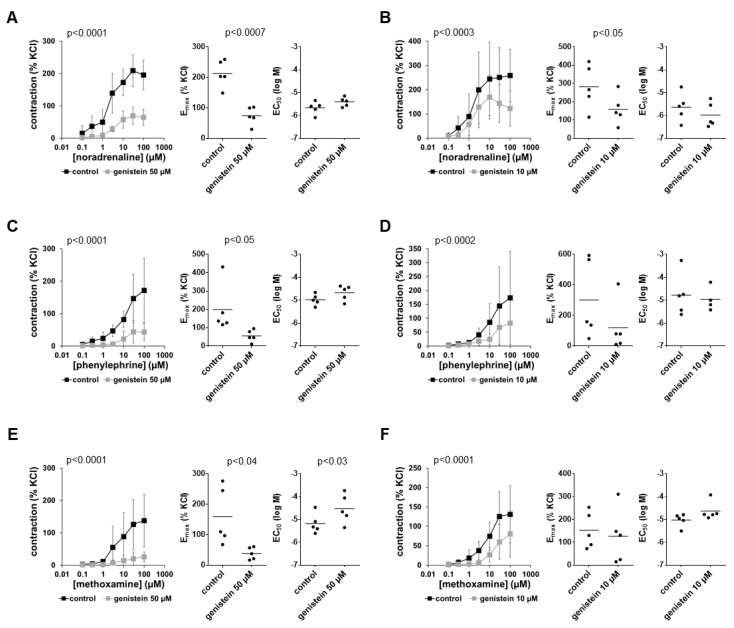
Effects of genistein on α_1_-adrenergic contractions of human prostate tissues. Contractions were induced by the α_1_-adrenergic agonists noradrenaline (**A**,**B**), phenylephrine (**C**,**D**) and methoxamine (**E**,**F**) in the presence of 50 µM genistein (**A**,**C**,**E**), 10 µM genistein (**B**,**D**,**F**) and corresponding amounts of solvent (DMSO) for the controls added 30 min before the construction of concentration–response curves. Data from experiments with tissues from *n* = 5 patients are shown in each diagram. In each experiment, tissue from one patient was allocated to the control and genistein groups. Data are means ± SDs in concentration–response curves (*p*-values for whole groups from two-way ANOVA), and E_max_ and EC_50_ values are from all single experiments (calculated by curve fitting) in scatter plots (*p*-values from two-tailed *t*-tests).

Noradrenaline, phenylephrine and methoxamine induced concentration-dependent contractions which were inhibited by 50 µM and 10 µM genistein (Figure 1). Contractions by all three α_1_-adrenergic agonists were inhibited by two-thirds or more using 50 µM genistein and by approximately half using 10 µM genistein (Figure 1). Inhibitions seen in concentration–response curves were reflected in reduced E_max_ values for agonists calculated by curve fitting (Figure 1). E_max_ values for noradrenaline were reduced by 66% (52 to 80) with 50 µM genistein (Figure 1A) and by 42% (16 to 67) with 10 µM genistein (Figure 1B) if values under genistein were normalized to values for the corresponding control groups in each single experiment. E_max_ values for phenylephrine were reduced by 68% (40 to 96) with 50 µM genistein (Figure 1C) and by 56% (23 to 89) with µM genistein (Figure 1D). E_max_ values for methoxamine were reduced by 73% (55 to 90) with 50 µM genistein (Figure 1E) and by 19% (−93 to 130) with 10 µM genistein (Figure 1F). Average EC_50_ values for noradrenaline and phenylephrine were elevated slightly, by less than half a magnitude, by 50 µM genistein, while average EC_50_ values for methoxamine were elevated by more than half a magnitude by 50 µM genistein (MD (logM) (0.13 to 1.18)) (Figure 1). Using 10 µM genistein, average EC_50_ values for all three α_1_-adrenergic agonists remained unchanged (Figure 1).

### 3.2. Effects of Genistein on EFS-Induced Contractions

**Figure 2:**
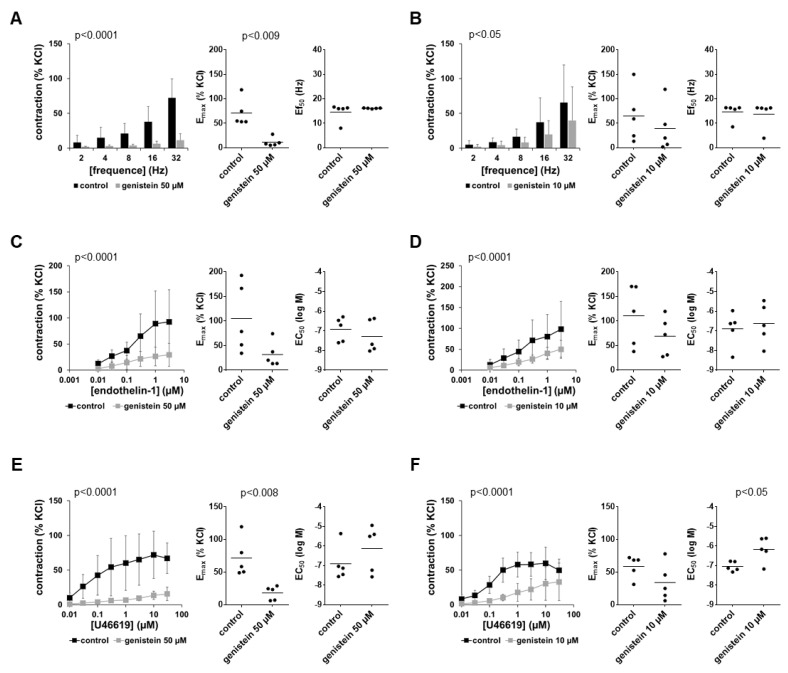
Effects of genistein on EFS-induced and non-adrenergic contractions of human prostate tissues. Contractions were induced by EFS (**A**,**B**), endothelin-1 (**C**,**D**) and U46619 (**E**,**F**) in the presence of 50 µM genistein (**A**,**C**,**E**), 10 µM genistein (**B**,**D**,**F**) and corresponding amounts of solvent (DMSO) for the controls added 30 min before the construction of frequency and concentration–response curves. Data from experiments with tissues from *n* = 5 patients are shown in each diagram. In each experiment, tissue from one patient was allocated to the control and genistein groups. Data are means ± SDs in frequency/concentration–response curves (*p*-values for whole groups from two-way ANOVA), and E_max_, Ef_50_ and EC_50_ values are from all single experiments (calculated by curve fitting) in scatter plots (*p*-values from two-tailed *t*-tests).

EFS induced frequency-dependent contractions which were inhibited by 50 µM and 10 µM genistein (Figure 2A,B). Inhibitions amounted to 75% or more at each frequency for 50 µM genistein and approximately half for 10 µM genistein (Figure 2A,B). Inhibitions seen in frequency response curves were paralleled by reduced E_max_ values, calculated by curve fitting, which decreased by 84% (69 to 98) with 50 µM and by 44% (−26 to 115) with 10 µM genistein (Figure 2A,B). Ef_50_ values remained unchanged by both concentrations of genistein (Figure 2A,B).

### 3.3. Effects of Genistein on Non-Adrenergic Contractions

Endothelin-1 and U46619 induced concentration-dependent contractions (Figure 2C–F). Contractions by both agonists were inhibited by two-thirds or more using 50 µM genistein and were still obvious using 10 µM genistein (Figure 2C–F). Inhibitions seen in concentration–response curves were reflected in reduced E_max_ values for agonists calculated by curve fitting (Figure 2C–F). E_max_ values for endothelin-1 were reduced by 65% (46 to 84) with 50 µM genistein (Figure 2C) and by 36% (18 to 54) with 10 µM genistein (Figure 2D). E_max_ values for U46619 were reduced by 74% (55 to 74) with 50 µM genistein (Figure 2E) and by 41% (−12 to 93) with 10 µM genistein (Figure 2F). EC_50_ values for endothelin-1 remained unchanged by both concentrations of genistein (Figure 2C,D). Average EC_50_ values for U46619 were increased by both concentrations of genistein; there were, however, large variations in the series using 50 µM genistein which amounted to more than half or nearly one magnitude (MD (logM) 0.78 (−1.63 to 3.19) with 50 µM, 0.88 (0.06 to 1.69) with 10 µM).

### 3.4. Effects of Daidzein on α_1_-Adrenergic Contractions

**Figure 3:**
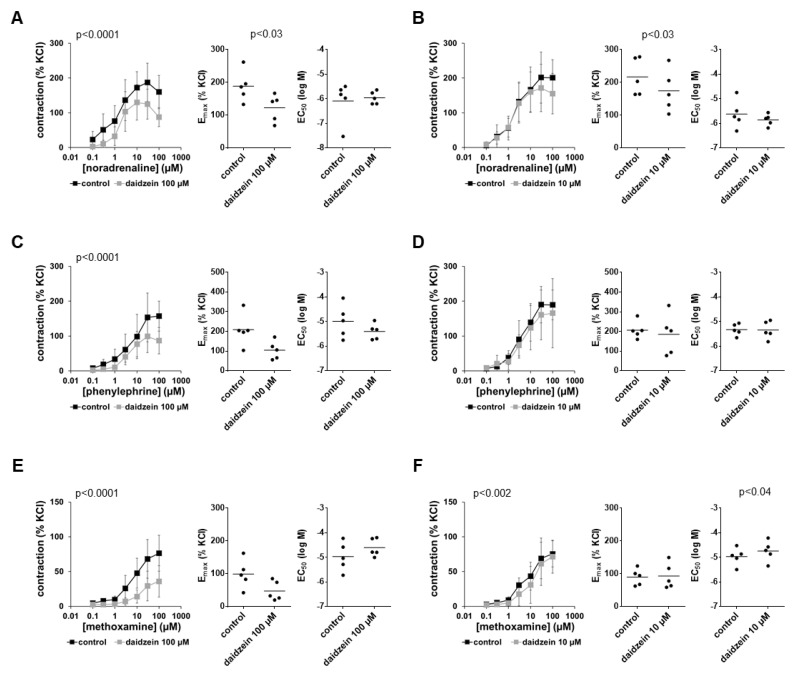
Effects of daidzein on α_1_-adrenergic contractions of human prostate tissues. Contractions were induced by the α_1_-adrenergic agonists noradrenaline (**A**,**B**), phenylephrine (**C**,**D**) and methoxamine (**E**,**F**) in the presence of 100 µM daidzein (**A**,**C**,**E**), 10 µM daidzein (**B**,**D**,**F**) and corresponding amounts of solvent (DMSO) added to controls 30 min before the construction of concentration–response curves. Data from experiments with tissues from *n* = 5 patients are shown in each diagram. In each experiment, tissue from one patient was allocated to the control and daidzein groups. Data are means ± SDs in concentration–response curves (*p*-values for whole groups from two-way ANOVA), and E_max_ and EC_50_ values are from all single experiments (calculated by curve fitting) in scatter plots (*p*-values from two-tailed *t*-tests).

Contractions induced by noradrenaline, phenylephrine and methoxamine were inhibited by 100 µM daidzein but not or to a neglectable extent by 10 µM daidzein (Figure 3). Inhibitions by 100 µM daidzein were largest at high agonist concentrations, where they amounted to around one-third for noradrenaline and phenylephrine, or around 50% for methoxamine (Figure 3). Inhibitions seen in concentration–response curves were paralleled by reduced E_max_ values (Figure 3), which decreased by 35% (10 to 59) for noradrenaline, 44% (12 to 76) for phenylephrine and 45% (2 to 87) for methoxamine with 100 µM daidzein. Average EC_50_ values were not consistently changed for all three agonists by 100 µM daidzein (Figure 3). Using 10 µM daidzein, E_max_ and EC_50_ values for all three α_1_-adrenergic agonists remained unchanged, apart from a decrease in E_max_ values for noradrenaline-induced contractions (Figure 3).

### 3.5. Effects of Daidzein on EFS-Induced Contractions

**Figure 4:**
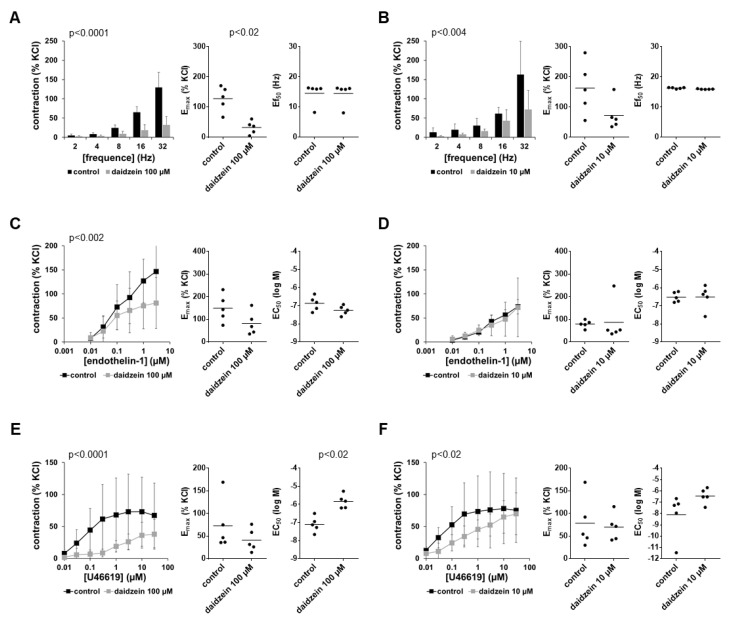
Effects of daidzein on EFS-induced and non-adrenergic contractions of human prostate tissues. Contractions were induced by EFS (**A**,**B**), endothelin-1 (**C**,**D**) and U46619 (**E**,**F**) in the presence of 100 µM daidzein (**A**,**C**,**E**), 10 µM daidzein (**B**,**D**,**F**) and corresponding amounts of solvent (DMSO) for the controls added 30 min before the construction of frequency and concentration–response curves. Data from experiments with tissues from *n* = 5 patients are shown in each diagram. In each experiment, tissue from one patient was allocated to the control and daidzein groups. Data are means ± SDs in frequency/concentration–response curves (*p*-values for whole groups from two-way ANOVA), and E_max_, Ef_50_ and EC_50_ values are from all single experiments (calculated by curve fitting) in scatter plots (*p*-values from two-tailed *t*-tests).

EFS-induced contractions were inhibited by 100 µM and 10 µM daidzein (Figure 4A,B). Inhibitions amounted to more than two-thirds at each frequency with 100 µM daidzein and up to 50% using 10 µM daidzein (Figure 4A,B). Inhibitions seen in frequency response curves were paralleled by reduced E_max_ values for EFS-induced contractions (Figure 4A,B), which were decreased by 70% (40 to 99) with 100 µM and by up to 50% (22 to 79) with 10 µM daidzein. Ef_50_ values remained unchanged by both concentrations of daidzein (Figure 4A,B).

### 3.6. Effects of Daidzein on Non-Adrenergic Contractions

Contractions induced by endothelin-1 were inhibited by 100 µM but not by 10 µM daidzein (Figure 4C,D). Inhibition was greatest at the two highest applied endothelin-1 concentrations, amounting to approximately 40% (Figure 4C). Inhibitions seen in concentration–response curves were paralleled by reduced E_max_ values for endothelin-1 (Figure 4C), which were decreased by 43% (1 to 84) with 100 µM daidzein. E_max_ values were not changed by 10 µM daidzein (Figure 4D), while Ef_50_ values remained unchanged with both concentrations of daidzein (Figure 4C,D).

Contractions induced by U46619 were inhibited by 100 µM and 10 µM daidzein (Figure 4E,F). Inhibitions were between more than two-thirds to half across wide ranges of concentration–response curves but were smaller or lacking at the highest applied agonist concentrations using 10 µM daidzein (Figure 4E,F). Inhibitions seen in concentration–response curves were reflected by E_max_ values for U46619, which were decreased by 39% (12 to 66) with 100 µM daidzein (Figure 4E) but remained unchanged with 10 µM daidzein (Figure 4F). Concentration–response curves for U46619 were right-shifted using both concentrations of daidzein, which was reflected in increased EC_50_ values for U46619 with daidzein (Figure 4E,F). As E_max_ values were not reduced with 10 µM daidzein, contractions in right-shifted concentration–response curves with daidzein fully recovered at high concentrations of U46619 (Figure 4F). EC_50_ values for U46619 were increased by more than one magnitude with both concentrations of daidzein (MD (logM) 1.27 (0.38 to 2.16) with 100 µM, 1.66 (−0.72 to 4.04) with 10 µM) (Figure 4E,F).

### 3.7. Effects of Combined Genistein and Daidzein on Contractions

**Figure 5:**
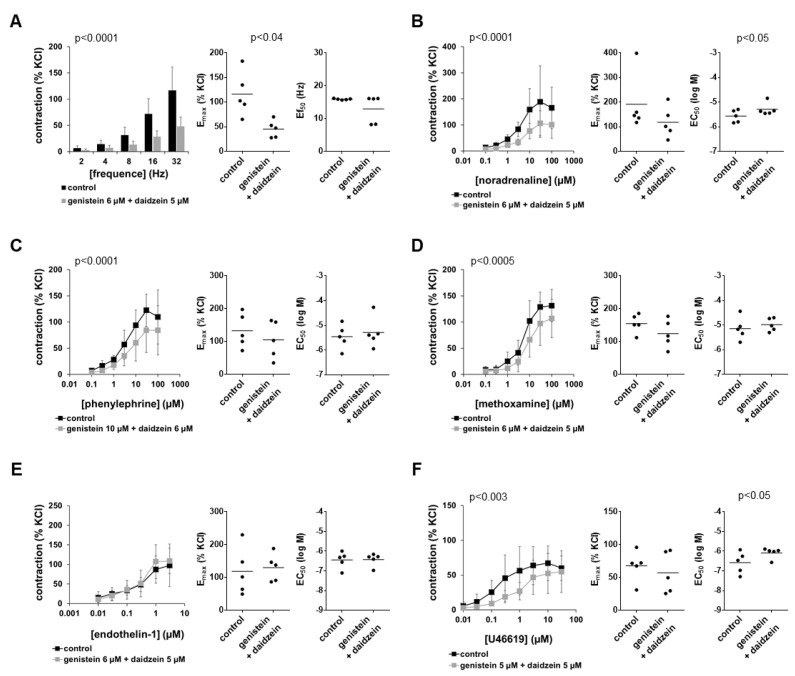
Effects of combined genistein and daidzein on EFS- and agonist-induced contractions of human prostate tissues. Contractions were induced by EFS (**A**), noradrenaline (**B**), phenylephrine (**C**), methoxamine (**D**), endothelin-1 (**E**) and U46619 (**F**) in the presence of 6 µM genistein plus 5 µM daidzein and corresponding amounts of solvent (DMSO) for the controls added 30 min before the construction of frequency and concentration–response curves. Data from experiments with tissues from *n* = 5 patients are shown in each diagram. In each experiment, tissue from one patient was allocated to the control and genistein + daidzein groups. Data are means ± SDs in frequency/concentration–response curves (*p*-values for whole groups from two-way ANOVA), and E_max_, Ef_50_ and EC_50_ values are from all single experiments (calculated by curve fitting) in scatter plots (*p*-values from two-tailed *t*-tests).

By combined application of 6 µM genistein and 5 µM daidzein and compared to the corresponding controls, EFS-induced contractions were inhibited by more than half at each applied frequency (Figure 5A). Inhibitions seen in frequency response curves were paralleled by reduced E_max_ values for EFS-induced contractions (Figure 5A), which were decreased by 57% (38 to 77). Ef_50_ values remained unchanged by the combination (Figure 5A).

Contractions by noradrenaline, phenylephrine and methoxamine were inhibited consistently but not by more than half by the combination of genistein with daidzein (Figure 5B–D). Inhibitions seen in concentration–response curves were paralleled by reduced E_max_ values for agonists, which were decreased by 32% (−17 to 80) for noradrenaline, 23% (−6 to 53) for phenylephrine and 19% (−15 to 54) for methoxamine (Figure 5B–D). EC_50_ values were increased slightly, i.e., less than half a magnitude for noradrenaline, or remained unaffected for phenylephrine and methoxamine (Figure 5B–D).

Contractions by endothelin-1 were not affected by the combination of 6 µM genistein and 5 µM daidzein (Figure 5E). U46619-induced contractions were inhibited partly, i.e., by around half at lower concentrations, but not or to a neglectable extent at high agonist concentrations (Figure 5F). Average E_max_ values for U46619 remained unaffected by the combination, while EC_50_ values were increased by around half a magnitude (MD (logM) 0.498 (0.03 to 0.97)) (Figure 5F).

### 3.8. Effects of Puerarin on α_1_-Adrenergic Contractions

**Figure 6:**
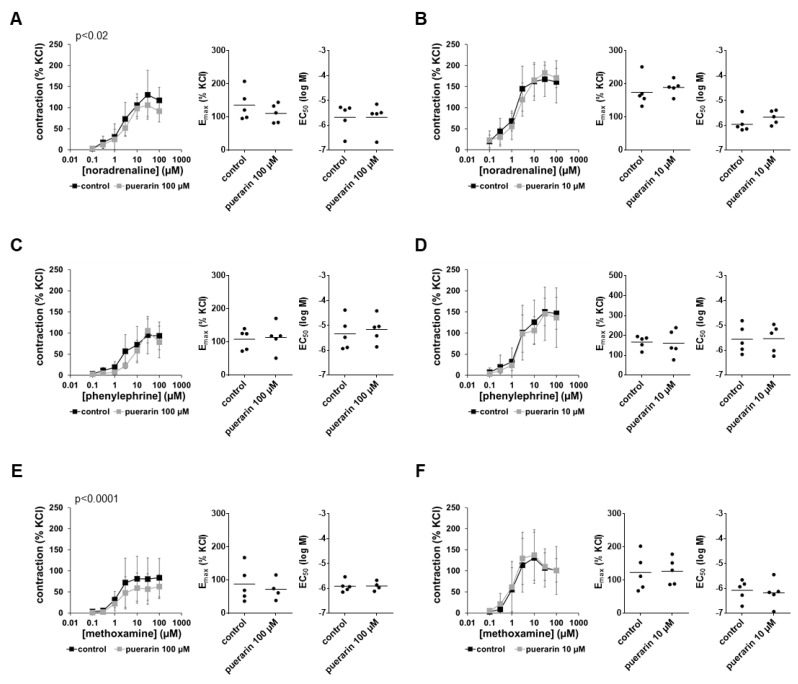
Effects of puerarin on α_1_-adrenergic contractions of human prostate tissues. Contractions were induced by the α_1_-adrenergic agonists noradrenaline (**A**,**B**), phenylephrine (**C**,**D**) and methoxamine (**E**,**F**) in the presence of 100 µM puerarin (**A**,**C**,**E**), 10 µM puerarin (**B**,**D**,**F**) and corresponding amounts of solvent (methanol) for the controls added 30 min before the construction of concentration–response curves. Data from experiments with tissues from *n* = 5 patients are shown in each diagram. In each experiment, tissue from one patient was allocated to the control and puerarin groups. Data are means ± SDs in concentration–response curves (*p*-values for whole groups from two-way ANOVA), and E_max_ and EC_50_ values are from all single experiments (calculated by curve fitting) in scatter plots (*p*-values from two-tailed *t*-tests).

Contractions induced by noradrenaline, phenylephrine and methoxamine were reduced marginally by 100 µM puerarin but not 10 µM puerarin (Figure 6). E_max_ values were neither substantially nor consistently changed by 100 µM puerarin and remained unaffected by 10 µM puerarin (Figure 6). EC_50_ values for all three α_1_-adrenergic agonists remained unchanged by both concentrations of puerarin (Figure 6).

### 3.9. Effects of Puerarin on EFS-Induced Contractions

**Figure 7:**
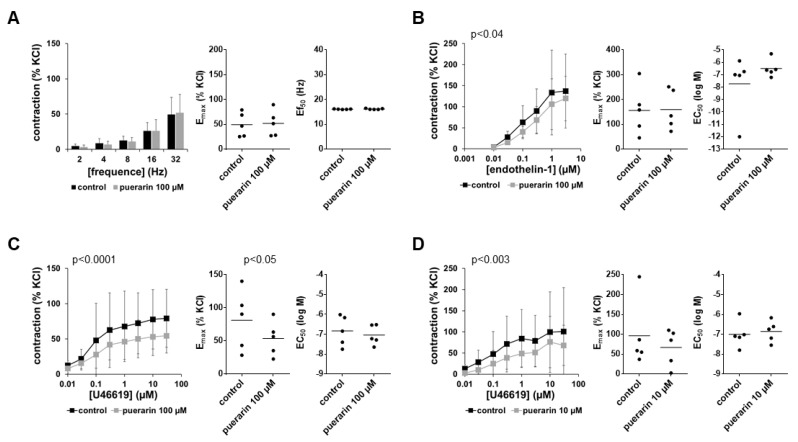
Effects of puerarin on EFS-induced and non-adrenergic contractions of human prostate tissues. Contractions were induced by EFS (**A**), endothelin-1 (**B**) and U46619 (**C**,**D**), in the presence of 100 µM puerarin (**A**–**C**) 10 µM puerarin (**D**) and corresponding amounts of solvent (methanol) for the controls added 30 min before the construction of frequency and concentration–response curves. Data from experiments with tissues from *n* = 5 patients are shown in each diagram. In each experiment, tissue from one patient was allocated to the control and puerarin groups. Data are means ± SDs in frequency/concentration–response curves (*p*-values for whole groups from two-way ANOVA), and E_max_, Ef_50_ and EC_50_ values are from all single experiments (calculated by curve fitting) in scatter plots (*p*-values from two-tailed *t*-tests).

Puerarin did not affect EFS-induced contractions using a concentration of 100 µM (Figure 7A), so effects at a lower concentration were not examined. According to the lack of effects in frequency response curves, no effects were seen in E_max_ and Ef_50_ values (Figure 7A).

### 3.10. Effects of Puerarin on Non-Adrenergic Contractions

Puerarin did not affect endothelin-1-induced contractions using a concentration of 100 µM (Figure 7B), so effects at a lower concentration were not examined. According to the lack of effects in concentration–response curves, no effects of puerarin were seen in E_max_ and EC_50_ values (Figure 7B).

Contractions induced by U46619 were inhibited by puerarin to similar extents at concentrations of 100 µM and 10 µM, with maximum decreases in contractions of around one-third (Figure 7C,D). Inhibitions were obvious in concentration–response curves and reflected in E_max_ values for U46619-induced contractions, which were decreased by 30% (16 to 44) with 100 µM puerarin and by 23% (−50 to 97) with 10 µM puerarin (Figure 7C,D). EC_50_ values for U46619 were not changed by puerarin (Figure 7C,D).

### 3.11. Effects of Genistein, Daidzein and Puerarin on the Proliferation of Stromal Cells

**Figure 8:**
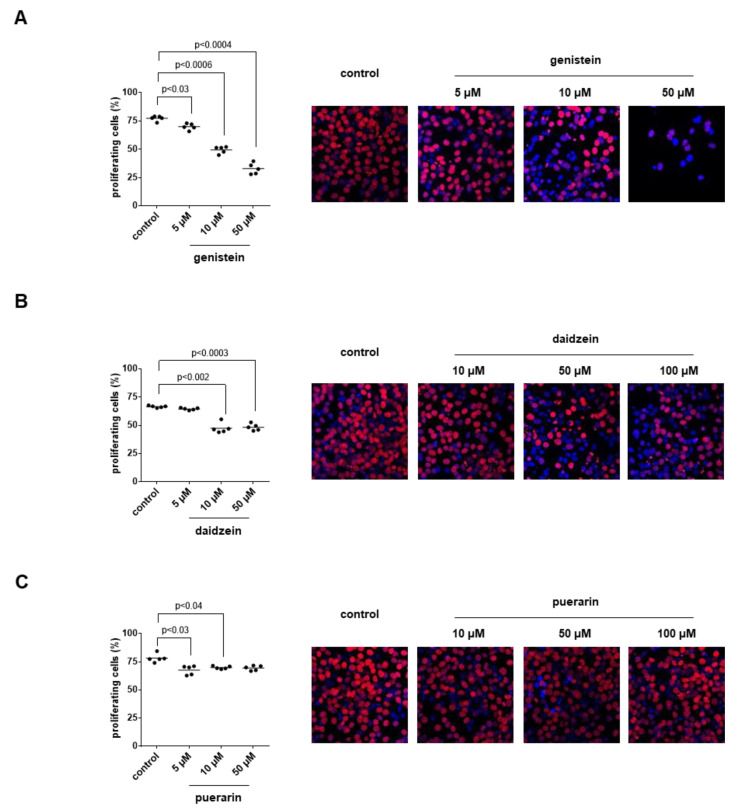
Effects of genistein, daidzein and puerarin on the proliferation of WPMY-1 cells. Proliferation was assessed by EdU assays (red, proliferating cells; blue, non-proliferating cells) after the cells were cultured for 24 h with genistein (**A**), daidzein (**B**) and puerarin (**C**) at the indicated concentrations or with solvent (DMSO for genistein and daidzein, methanol for puerarin) for the controls. Shown are all single values together with means from the quantification of proliferating cells in five independent experiments, along with representative images for each series, and *p*-values from comparisons with values for the corresponding DMSO group determined by one-way ANOVA with Dunnett’s tests.

Proliferation of WPMY-1 cells, assessed by EdU assays, was concentration-dependently decreased by genistein (Figure 8A). Decreases in proliferation rates amounted to 9% (4 to 15) with 5 µM, 36% (29 to 43) with 10 µM and 57% (48 to 67) with 50 µM genistein if proliferation rates with genistein were referred to corresponding controls without genistein in each single experiment. Daidzein reduced the proliferation of WPMY-1 cells as well and to similar extents at the two highest applied concentrations (Figure 8B). Decreases in proliferation rates amounted to 3% (0 to 6) with 5 µM, 29% (21 to 37) with 10 µM daidzein and 27% (22 to 32) with 50 µM daidzein. Puerarin decreased the proliferation of WPMY-1 cells only slightly and not concentration-dependently (Figure 8C), resulting in decreases in proliferation of 13% (6 to 21) with 5 µM, 11% (4 to 17) with 10 µM and 11% (3 to 19) with 50 µM puerarin.

### 3.12. Effects of Genistein, Daidzein and Puerarin on Apoptosis and Cell Death of Stromal Cells

**Figure 9:**
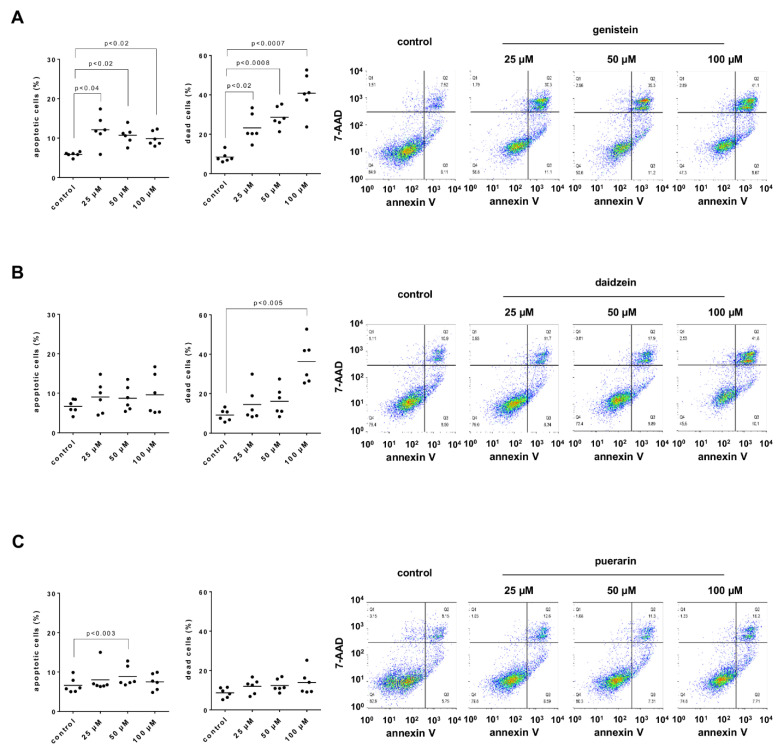
Effects of genistein, daidzein and puerarin on apoptosis and cell death in WPMY-1 cells. Numbers of cells in apoptosis (annexin V-positive, 7-AAD-negative) and of dead cells (resulting from apoptosis and/or necrosis; annexin V-positive, 7-AAD-positive) were assessed by flow cytometry. Flow cytometry was performed following culture with genistein (**A**), daidzein (**B**) and puerarin (**C**) at the indicated concentrations or solvent (DMSO for genistein and daidzein, methanol for puerarin) for the controls for 24 h. Shown are all single values together with means from the quantification of five independent experiments and representative experiments for each series, along with *p*-values from one-way ANOVA with Dunnett’s tests.

Genistein increased the numbers of apoptotic and dead WPMY-1 cells, which were assessed by flow cytometric analysis of 7-AAD and annexin V (Figure 9A). Increases in the numbers of dead cells but not numbers of apoptotic cells by genistein were concentration-dependent (Figure 9A). The numbers of apoptotic cells were 2.1-fold higher (1.2 to 3.0) than for the corresponding controls with 25 µM, 1.9-fold higher (1.3 to 2.5) with 50 µM and 1.7-fold higher (1.3 to 2.2) with 100 µM genistein if the numbers with genistein were referred to the corresponding controls without genistein in each single experiment. The numbers of dead cells were 4.0-fold higher (2.8 to 5.2) than for the corresponding controls with 25 µM, 4.9-fold higher (4.3 to 5.4) with 50 µM and 7.0-fold higher (5.3 to 8.7) with 100 µM genistein.

Daidzein increased the numbers of apoptotic WPMY-1 cells without a concentration-dependent relationship and concentration-dependently increased the numbers of dead cells, which were most pronounced with the highest applied concentration (Figure 9B). The numbers of apoptotic cells were 1.5-fold higher (0.9 to 2.2) than for the corresponding controls with 25 µM, 1.5-fold higher (1.0 to 1.9) with 50 µM and 1.6-fold higher (0.8 to 2.4) with 100 µM daidzein. The numbers of dead cells were 2.6-fold higher (0.6 to 4.6) than for the corresponding controls with 25 µM, 2.8-fold higher (1.2 to 4.5) with 50 µM and 6.4-fold higher (3.7 to 9.0) with 100 µM daidzein.

Puerarin increased the numbers of apoptotic and dead WPMY-1 cells only slightly or to a neglectable extent, without concentration-dependent relationships (Figure 9C). The numbers of apoptotic cells were 1.4-fold higher (0.9 to 1.9) than for the corresponding controls with 25 µM, 1.6-fold higher (0.9 to 2.2) with 50 µM and 1.3-fold higher (0.9 to 1.7) with 100 µM puerarin. The numbers of dead cells were 2.1-fold higher (1.3 to 2.8) than for the corresponding controls with 25 µM, 2.2-fold higher (1.4 to 2.9) with 50 µM and 2.5-fold higher (0.9 to 4.0) with 100 µM puerarin.

### 3.13. Effects of Genistein, Daidzein and Puerarin on the Viability of Stromal Cells

**Figure 10:**
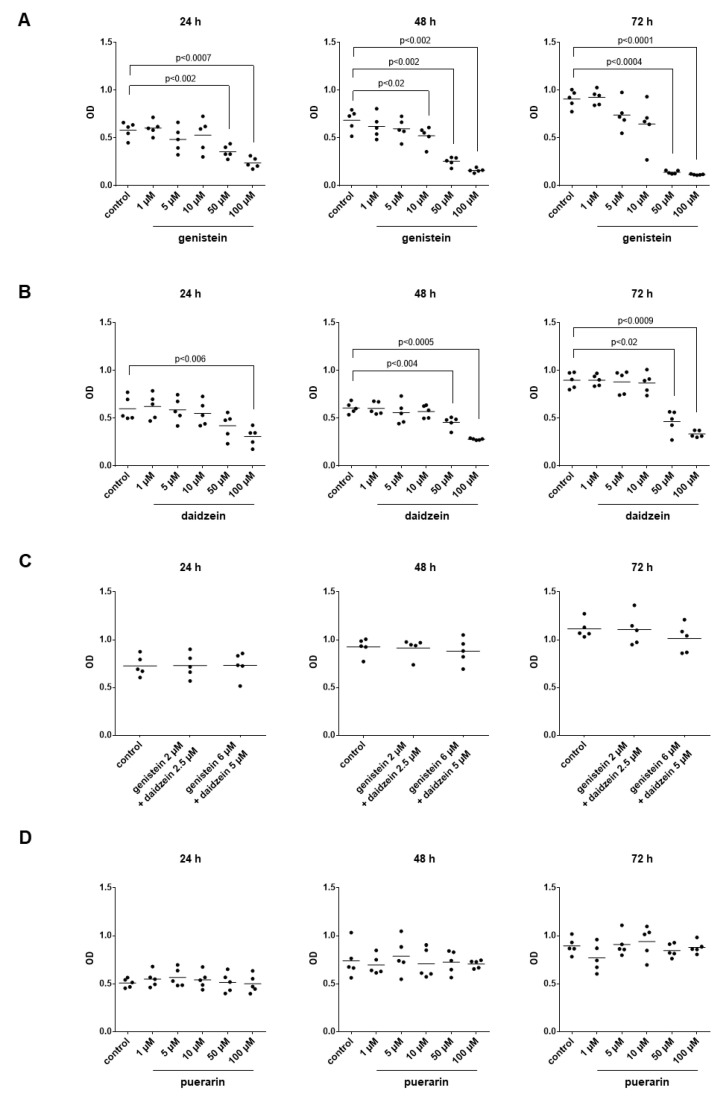
Effects of genistein, daidzein and puerarin on the viability of WPMY-1 cells. Viability was assessed by CCK-8 assays, following culture with genistein (**A**), daidzein (**B**), combinations of genistein with daidzein (**C**) and puerarin (**D**) at the indicated concentrations or solvent (DMSO for genistein and daidzein, methanol for puerarin) for the controls and for indicated periods. Shown are all single values together with means from quantification of five independent experiments and representative experiments for each series, along with *p*-values from one-way ANOVA with Dunnett’s tests.

Genistein reduced the viability of WPMY-1 cells, which was assessed by CCK-8 assays 24 h, 48 h and 72 after the addition of isoflavones (Figure 10A). The viability, as well as the effects of genistein, increased over time (Figure 10A). Decreases in viability started with 5 µM, without exceeding decreases of 50% up to 10 µM but approaching decreases of 59% (51 to 67) after 24 h and of 87% (86 to 89) after 72 h with 100 µM genistein if values with genistein were referred to the corresponding controls without genistein in each single experiment (Figure 10A).

Daidzein decreased the viability of WPMY-1 cells, which was concentration-dependent but started with a concentration of 50 µM, while no certain decreases occurred with lower concentrations (Figure 10B). Maximum decreases amounted to 49% (33 to 64) after 24 h, 54% (50 to 58) after 48 h and 62% (55 to 70) after 72 h with 100 µM daidzein (Figure 10B).

The combination of 2 µM genistein with 2.5 µM daidzein did not affect the viability of WPMY-1 cells after 24–72 h (Figure 10C). The combination of 6 µM genistein with 5 µM daidzein did not affect the viability after 24–48 h, while the viability was reduced by 9% (−0.3 to 19) after 72 h with this combination (Figure 10C).

Puerarin did not affect the viability of WPMY-1 cells across the examined concentration range, with a maximum concentration of 100 µM puerarin (Figure 10D).

## 4. Discussion

High isoflavone exposure due to soy-rich nutrition correlated with low prevalence of BPH and voiding symptoms in epidemiological studies [12,13,14]. Clinical observations confirmed possible effects of isoflavones on BPH/LUTSs, as preparations from isoflavone-rich plants partly reduced symptoms and the risk for symptomatic BPH [37]. However, previous experimental studies addressing isoflavone impacts in the prostate focused on estrogenic effects, hormone-disrupting properties, epithelial prostate cells, animal models and prostate cancer [38,39,40,41,42,43]. Consequently, the effects of isoflavones on human prostate smooth muscle contraction and on the growth of stromal cells are mostly unknown, and directed comparisons to identify the most active isoflavones in the prostate are still pending. Our present findings suggest that genistein inhibited human prostate smooth muscle contractions to a remarkable extent and had marked effects on growth-related functions of stromal cells, whereas the effects of daidzein were weaker and the effects of puerarin appeared overall limited. Our findings provide a new basis for understanding isoflavone effects on voiding symptoms and BPH, which are relevant in light of the expanding popularity of soy food seen in many regions and the availability of isoflavone-rich plant preparations as dietary supplements.

We observed inhibition of neurogenic-, α_1_-adrenergic-, endothelin-1- and U46619-induced contractions of human prostate tissues by genistein. Inhibitions with 50 µM of genistein approached the effects of α_1_-adrenoceptor antagonists on EFS-induced and α_1_-adrenergic contractions of human prostate tissues previously observed by us under similar conditions [44]. Using 10 µM, the effects were still obvious, although weaker. In addition, genistein inhibited contractions induced by U46619 and endothelin-1 to a similar degree as α_1_-adrenergic contractions. These non-adrenergic contractions are resistant to α_1_-adrenergic antagonists but may induce a full, maximum prostate smooth muscle tone [21]. In BPH, increased prostate smooth muscle tone may contribute to urethral obstruction and finally to voiding symptoms [16,18]. α_1_-blockers are the first line option for medical treatment and are supposed to improve symptoms by inhibition of α_1_-adrenergic prostate smooth muscle contractions [16,18]. However, their efficacy to improve voiding symptoms is limited, probably due to contractions by non-adrenergic mediators [21,22]. Non-adrenergic contractions may keep prostate tone elevated and account for medication-refractory LUTSs, so that compounds showing inhibitory effects on non-adrenergic contractions are potentially of high clinical interest [21,22]. To the best of our knowledge, previous findings addressing the effects of genistein on prostate smooth muscle contractions are limited to one series, demonstrating the inhibition of EFS-induced contractions in rat prostate tissues with 100 µM genistein [41], while data for agonist-induced contractions, lower concentrations or human tissues are not available.

In cultured stromal cells, we observed concentration-dependent inhibition of proliferation, increases in cell death and decreases in viability with genistein, paralleled by concentration-independent increases in apoptosis. Cell death occurred probably independently of apoptosis, as the rate of apoptotic cells was insufficient to explain higher percentages of dead cells. Thus, genistein may act on different growth-related functions of stromal cells, including proliferation, apoptosis and apoptosis-independent survival, resulting in the observed decreases in viability starting at genistein concentrations of 5–10 µM. Previous studies addressing effects of genistein on stromal cells are limited to one series, where a single concentration of 10 µM showed no effect on survival in the presence of androgen [45]. For glandular epithelial cells of the prostate, antiproliferative and apoptotic effects of genistein are well-documented [46,47,48]. In BPH, both cell types may contribute to epithelial, stromal or mixed hyperplasia and thus to prostate growth and urethral obstruction [17]. Our current findings, together with previous findings from epithelial cells, may suggest that genistein could reduce prostate growth in BPH in vivo, provided adequate concentrations occur in the prostate. In fact, genistein inhibited prostate growth in rodent models of testosterone-induced BPH and high-fat-induced prostate growth [38,40,43], as well as ex vivo growth in prostate tissues from patients with BPH [49].

Similar to genistein, isoflavone-rich plant preparations reduced experimentally induced and age-related prostate growth in in vivo studies on rodents, including extracts from red clover, black soybeans, Kudzu (*Pueraria montana*) and *Pueraria mirifica* [38,40,42,50,51], as did a soy-based, phytoestrogen-rich diet [39]. These and similar preparations contain several different isoflavones, with compositions and predominant compounds varying between plant species. In soybeans, genistein and daidzein belong to the predominant isoflavones, with the content of genistein ranging 1.5–2-fold higher than daidzein [15,39]. Similar to animal models, preparations from soy and red clover reduced prostate size and slightly improved voiding symptoms in humans [37,52], which may explain the widely assumed benefits of soy-rich nutrition in the context of BPH and voiding symptoms. Clinically, voiding symptoms in BPH are expressed by the maximum urinary flow rate (Q_max_) and the international prostate symptoms score (IPSS) assessed by standardized questionnaires, while postvoid residual urine volume (PVR) allows estimation of the risk for progression. In patients with BPH with a Q_max_ < 15 mL/s, an IPSS > 8 and a PVR > 150 mL, daily treatment with a soy-based preparation for one year containing 40 mg isoflavones increased Q_max_ by 1.7 mL/s, reduced IPSS by 4 points and decreased PVR by 49 mL [37]. However, these were not different from placebo effects: increases in Q_max_ by 1.3 mL/s, decreases in IPSS by 2.9 points and decreases in PVR by 40 mL [37]. In another single-arm trial without a placebo group, patients with BPH received a red clover extract for one year equivalent to 60 mg of isoflavones per day, resulting in a decrease in prostate volume of 10% and a decrease in IPSS of 1.2 points [52]. In general, improvements by medical interventions require decreases in IPSS by 3 or more points in order to be perceptible by patients [53]. No threshold values have been defined for clinically relevant improvements in Q_max_, as this highly depends on the baseline Q_max_ before treatment initiation. In large-scale trials, α_1_-blockers enhanced Q_max_ by 0.7–2.5 mL/s, which has been considered clinically relevant [54,55].

Consequently, the available clinical data and the effects of these preparations are both limited but may reflect a higher effectiveness of soy compared to red clover. The composition of isoflavone pools may differ between species. Genistein and daidzein are predominant isoflavones in soybeans and red clover and constitute most parts of their total isoflavone content [1,4,15]. However, the availability and content of genistein and daidzein in soy-based food may vary and depend on processing, including fermentation and washing procedures, even though they are high overall [15]. The predominant isoflavone in *Pueraria* roots is puerarin, with levels exceeding those of genistein plus daidzein together by around five-fold and contributing the largest part of the total isoflavone content [1,4,8]. In contrast, puerarin was undetectable in isoflavone preparations from soybeans and red clover [1,4]. Our findings suggest that genistein may be the isoflavone contributing most to the beneficial effects of mixed isoflavone preparations for BPH and LUTSs. Even though daidzein caused obvious inhibitions of EFS- and agonist-induced contractions and showed growth-inhibiting effects in stromal cells, these effects were weaker compared to 50 µM genistein and were minor or lacking at a concentration of 10 µM. Puerarin, the most abundant isoflavone in *Pueraria* roots, did not inhibit smooth muscle contractions of human tissues, apart from partial inhibition of U46619-induced contractions, while growth-related functions of stromal cells were widely unaffected. Regarding the cardiovascular system, effects of vasodilation and relaxation of vascular smooth muscle due to puerarin are well-known [11], and antihypertensive effects of the roots of *Pueraria* species used in Eastern Asian medicine have been considered [56]. In line with the lack of effects observed for puerarin in our study, improvements in voiding symptoms by *Pueraria* preparations have never been noted, despite their widespread use in traditional medicine in Eastern Asia and as dietary supplements in Western nations [8,9].

Isoflavone concentrations in the prostate have been assessed in patients receiving isoflavone preparations in several trials. Concentrations in the prostate were found to be higher compared to the plasma following the use of isoflavone preparations. In patients with BPH receiving 112.5 mg/d isoflavones, composed of 65.7% genistein and 31.7% daidzein in commercially available capsules, for three days, genistein concentrations amounted to 0.58 nmol/g prostate tissue but 0.78 µM in the plasma, and daidzein concentrations were 0.47 nmol/g prostate tissue but 0.66 µM in the plasma [57]. Assuming this could be simply extrapolated, this may correspond to 5.8 µM genistein or 4.7 µM daidzein in the prostate. Similar values were reported for patients undergoing radical prostatectomy for prostate cancer who received 27.2 mg of isoflavones (containing 10.6 mg genistein and 13.3 mg daidzein) per day in a soy-based preparation for 12–14 days, resulting in genistein concentrations of 2.2 µmol/kg in prostate tissue but 0.45 µM in the plasma, and daidzein concentrations of 2.4 µmol/kg in tissue but 0.28 µM in the plasma [58]. In men consuming a soymilk-based beverage for seven days containing 42–60 mg isoflavones, both compounds were detectable in the prostate fluid (consisting mostly of prostate secretion), with concentrations between 0.2 and 1 µM for genistein and between 0.2 and 30 µM for daidzein [48]. Consequently, we examined the effects of a combination of 6 µM genistein and 5 µM daidzein, representing a composition and concentration which may be expected from isoflavone-rich preparations in the prostate. This combination still inhibited α_1_-adrenergic-, EFS- and U46619-induced contractions. Whether these effects are sufficient for urodynamic effects in vivo cannot be concluded from our findings but it appears possible that they are. It could be speculated that even slight inhibitions of adrenergic and non-adrenergic contractions and slight growth-inhibiting effects may add up, which cannot be expected from α_1_-blockers.

While isoflavone concentrations in the prostate obviously exceed the plasma concentrations after ingestion of isoflavone preparations, the bioavailability and activity of isoflavones depend on glycosylation, conjugation and metabolization, cofactors and other nutrients, duration and form of ingestion, and other variables [6,59,60]. The naturally occurring form of isoflavones, predominating in plants and ingested with soy food are glucosides, which are poorly absorbed [6,59]. The absorbed forms are aglycones, resulting from hydrolysis of the sugar moiety by bacterial and intestinal mucosal β-glucosidases [6,59]. Accordingly, the bioavailability is higher with fermented soy products, for which the content of aglycones is higher, compared to non-fermented soy products [6]. Furthermore, deglycosylation and absorption are critically enhanced by fiber-rich, matrix food components [59,60]. Following absorption, aglycosylated isoflavones undergo conjugation, mainly including glucuronidation but also sulfation, other conjugations and further metabolization [6,59]. Conjugation occurs mostly in enterocytes, and glucuronides are the major forms of isoflavones in the plasma, where the free, unconjugated forms represent only a small portion [6].

Metabolites of isoflavones may be bioactive as well, with equol resulting from the conversion of daidzein as a prominent example [59]. However, our study focused on non-conjugated isoflavone compounds and did not include metabolites or modified forms. Apart from food matrix components, absorption and bioavailability but also bioactivity in vitro may depend on further cofactors and nutrients. In cultured prostate cancer cells, isoflavones show antiproliferative and antigenotoxic effects, which are enhanced by metal ions, in particular iron, copper and zinc ions [61]. In fact, the risk for prostate cancer incidence decreases with soy food consumption, which is not related to soy protein but has been referred to isoflavones [62]. The mortality from prostate cancer, however, is not reduced by isoflavones or may even increase [62,63], reflecting the need for an improved understanding of still incompletely understood isoflavone effects in the prostate and of their relationships with cofactors and other nutrients.

Considering that genistein showed the largest effects of three different isoflavones on prostate functions, the use of genistein rather than mixed isoflavone preparations for the treatment of voiding symptoms in BPH could be discussed. In contrast to available drugs, genistein may theoretically address contraction and growth in the prostate at once, as a single compound. Currently, this requires combination therapies [18]. Several compounds were recently identified as inhibiting prostate smooth muscle contraction and stromal cell growth at once, similar to genistein. However, these were inhibitors for kinases of GTPases, with limited translational value due to expectable side effects. Considering that genistein is a plant constituent and that pure genistein is available without prescription as a dietary supplement, it may be easily assumed that genistein is better tolerated than most small molecule inhibitors, though this may be in fact a dangerous oversimplification [64]. Genistein belongs to the group of “pan-assay interference compounds” (PAINS), together with other natural phytochemicals [65], showing low specificity, multiple and uncertain targets and a plethora of effects in preclinical studies. In fact, genistein is applied as an “phytoestrogenic” to reduce menopause symptoms [6]. In experimental medicine, it has been used as an inhibitor for non-receptor, Src family tyrosine kinases, so that it appears surprising that the compound is freely available as a dietary supplement. While it is apparently safe in women using it against menopause symptoms, tolerability is unknown in elderly men with BPH and voiding symptoms, and side effects should not be excluded. Isoflavones are potent inhibitors of cytochrome P450 isoforms and modulate activities of various other metabolic enzymes and transporters. As a result, they may not only influence their own bioavailability but also strongly influence the pharmacokinetics and dispositions of co-administered drugs. Clinically relevant and even life-threating drug–herb, drug–diet and drug–drug interactions have been reported due to the use of isoflavones [64]. Thus, the safety of genistein and isoflavones needs to be assessed before any application in BPH, where polypharmacy is common.

## 5. Conclusions

The major soy isoflavones, genistein and daidzein, exert sustained effects on prostate smooth muscle contractions and stromal cell growth, which may explain the inverse relationships between soy-rich nutrition, BPH and voiding symptoms. Improvements in voiding symptoms due to isoflavone-rich preparations with soy or red clover appear possible.

## Data Availability

The datasets supporting the conclusions of this article are included within the article.
